# Factors Explaining the Use of Web-Based Consultations With Physicians by Young and Middle-Aged Individuals in China: Qualitative Comparative Analysis

**DOI:** 10.2196/50036

**Published:** 2024-03-29

**Authors:** Chunyu Zhang, Ning Hu, Rui Li, Aiping Zhu, Zhongguang Yu

**Affiliations:** 1 Department of Human Resources China-Japan Friendship Hospital Beijing China; 2 School of Management Beijing University of Chinese Medicine Beijing China; 3 Respiratory Centre China-Japan Friendship Hospital Beijing China; 4 Hospital Office China-Japan Friendship Hospital Beijing China

**Keywords:** web-based consultation, Andersen Behavioral Model, qualitative comparative analysis, perceived convenience, complementary role, user's confidence, China

## Abstract

**Background:**

It was only upon the occurrence of the COVID-19 pandemic that the demand for web-based consultations with physicians grew at unprecedented rates. To meet the demand, the service environment developed rapidly during the pandemic.

**Objective:**

This study aimed to identify the current status of the use of web-based consultations with physicians among young and middle-aged Chinese individuals and explore users’ perspectives on key factors that influence its use in terms of optimizing benefits and compensating for disadvantages.

**Methods:**

We conducted semistructured interviews with 65 individuals (aged 18 to 60 years) across China between September and October 2022. The interviewees were selected through snowball sampling. They described their experiences of using web-based physician consultations and the reasons for using or not using the service. Based on the Andersen Behavioral Model, a qualitative comparative analysis was used to analyze the factors associated with the use of web-based physician consultations and explore the combinations of these factors.

**Results:**

In all, 31 (48%) of the 65 interviewees used web-based consultation services. The singular necessary condition analysis revealed that the complementary role of the service and perceived convenience are necessary conditions for the use of web-based consultation services, and user’s confidence in the service was a sufficient condition. Based on the Andersen Behavioral Model, the configuration analysis uncovered 2 interpretation models: an enabling-oriented model and a need-oriented model. The basic combination of the enabling-oriented model included income and perceived convenience. The basic combination of the need-oriented model included complementary role and user’s confidence.

**Conclusions:**

Among the factors associated with the use of web-based consultations, perceived convenience, complementary role, and user’s confidence were essential factors. Clear instructions on the conduct of the service, cost regulations, provider qualifications guarantee, privacy and safety supervision, the consultations’ application in chronic disease management settings, and subsequent visits can promote the positive development of web-based consultations.

## Introduction

The term “internet health care service” refers to a closed-loop service that includes health education, medical information inquiry, electronic health files, disease risk assessment, web-based consultation with physicians, electronic prescription, remote consultation, and remote treatment and rehabilitation via the internet and other technological means [1].

The COVID-19 pandemic created a demand for internet health care services at an unprecedented rate [2-4], as patients became reluctant to go to hospitals because of the fear of infection [5,6]. Accordingly, an increasing number of hospitals and internet companies started to, and continue to, venture into the internet health care industry. Reports show that 52% of outpatient departments in Germany have already adopted internet health care services [7]. By June 2022, more than 1700 hospitals in China were providing services using the internet, an increase from 100 in December 2018 [8].

These rapid changes and the quick adoption of internet health care services during the pandemic, however, have impeded the possibility of sufficient analyses on the experience of accessing these services and on how providers can complement the functions of these services to make them more accessible and attractive to users, as well as promote patients’ intention to use the service.

In this study, we only focus on web-based consultations with physicians, which is a core and controversial segment of internet health care services. Some researchers have studied the barriers to and facilitators of web-based consultations and found that perceived convenience, emotional preference, perceived risks, etc, influence behavioral intention [9,10].

However, factors associated with the use of web-based consultations are mixed. Understanding which factors are essential is conducive to optimizing benefits and compensating for disadvantages. Given that young (18-35 years) and middle-aged (35-60 years) individuals are the groups that use web-based consultations the most frequently, we conducted interviews among them to explore the reasons why web-based consultations are used or not used. Then, based on the Andersen Behavioral Model, we applied a qualitative comparative analysis (QCA) approach to analyze evidence from the interviews, to identify how combinations of these interdependent factors lead to the use of web-based consultations with physicians.

## Methods

### Theoretical Background

The Anderson Behavioral Model, developed by Andersen [11] in 1968, has been widely used to analyze the factors associated with health service use based on 3 dimensions: predisposing, enabling, and need factors [12-14]. Based on the Andersen Behavioral Model, this study also discussed the factors affecting web-based consultations with physicians in China.

### QCA Methodology

The use of web-based consultation has complex influences rather than a single effect. QCA has been applied to explore the different combinations of health care interventions because it bridges qualitative and quantitative methodologies [15]. Based on set theory, QCA compares characteristics of the cases in relation to the outcomes by a scoring system. Moreover, QCA has an advantage in analyzing small samples, which usually requires 10 to 80 cases [16,17]. Crisp-set QCA (csQCA) yields binary scores of 0 and 1, indicating “full out” or “full in” in certain conditions [18].

### Sample Selection and Data Collection

The semistructured interviews were centered around three broad questions: (1) Do you have experience using internet health care services? (2) If yes, which function do you use and why do you use it? Which function do you never use and why are you are reluctant to use it? and (3) If no, why do you never use internet health care services? When describing their experiences, the participants were asked to share examples and not only feelings about internet health care services.

We conducted interviews with residents of provinces in Eastern, Western, and Central China between September and October 2022.

The initial 5 samples were selected by convenience, and they were patients visiting the China-Japan Friendship Hospital. Subsequently, we asked them to recommend 1 or 2 interviewees, such as their friends, colleagues, or relatives, randomly. We repeated this process until the information on why internet health care services were or were not used was saturated. To obtain representative samples, we analyzed the characteristics of former samples and provided detailed requirements with regard to age, location, income, education, and sex for the following samples.

In total, 70 participants were interviewed, and 5 interviews were excluded owing to a lack of information regarding web-based consultations with physicians. The sample size for QCA should be at least 2*^k^*, where *k* is the number of conditions [17,19]. The study includes 6 conditions; hence, the sample size should be at least 64. Ultimately, the study included 65 interviews.

### Variable Measurement and Calibration

We analyzed the transcripts using a team-based inductive approach. First, the audio data were transcribed verbatim by a third-party company specialized in transcriptions in the Chinese language; once transcribed, the audio recordings were subsequently discarded to protect the participants’ confidentiality. Second, the first round of open coding was conducted using NVivo 12 (QSR International), and we coded the transcripts independently. We discussed and resolved discrepancies and then recoded the data to compile the major themes. Finally, based on the Andersen Behavioral Model, both the conditions and the results were identified by the lead author, reviewed by coauthors, and finalized by the corresponding author (see Table 1). In this study, csQCA was conducted using a program for crisp and fuzzy set with the *fsQCA3.0* package (Charles C Ragin and Sean Davey).

**Table 1:** Variables in the Andersen Behavioral Model of health service use and its measurements.

Dimensions of the Andersen Behavioral Model and variables			Measurement condition
**Predisposing**			
	Age	0 = 18 to <35 years old 1 = 35-60 years old	
	Education	0 = Master’s and doctorate degree 0.5 = Bachelor’s degree 1 = Below bachelor’s degree	
**Enabling**			
	Annual income	0 = <CN ¥120,000 (<US $16,804) 1 = ≥CN ¥120,000 (≥US $16,804)	
	Perceived convenience	0 = “Complex conduct procedure” and “late response” 1 = “Time saving” and “avoiding infection”	
**Need**			
	Complementary role	0 = “Unable to perform physical examination or laboratory test,” “compared with face-to-face consultation, less smooth communication,” and “uncovered by social health insurance” 1 = “Providing primary suggestion” and “handling the minor problems”	
	User’s confidence	0 = “Doubts about the safety and privacy of the platforms, and about the qualification of the doctor” 1 = There is no doubt	
**Use**			
	Web-based consultation	0 = Never used web-based consultation 1 = Has experience in using web-based consultation	

### Ethical Considerations

This study was approved by the China-Japan Friendship Hospital (approval 202-ky-032). We asked the participants whether they would be willing to be interviewed over the phone. Once the participants confirmed that they were interested in participating in the study, we made an appointment with them before the interview. At the beginning of the interview, we reviewed a consent form with the participants and obtained their verbal consent to proceed. Interviews were conducted primarily through phone calls because we aimed to reach more residents from different regions across China. All interviews were recorded and transcribed verbatim for data analysis. No compensation was provided for participation.

## Results

### Participants’ Characteristics

Participants were recruited from the provinces of Beijing, Shanghai, Guangdong, and Zhejiang in Eastern China (31/65, 48%); Jilin, Henan, and Jiangxi in Central China (14/65, 22%); and Sichuan, Yunan, and Qinghai in Western China (20/65, 31%). In total, 38% (25/65) of the participants were male and 62% (40/65) were female, and the participants’ average age was 35.4 (range 18-51) years (Table 2).

**Table 2:** Demographic characteristics of the participants (n=65).

Characteristics		Using web-based consultation, n (%)	Not using web-based consultation, n (%)	Chi-square (*df*)		*P* value	
**Sex**					0.302 (1)		.58
	Male (n=25)	13 (52)	12 (48)				
	Female (n=40)	18 (45)	22 (55)				
**Age range (years)**					1.046 (1)		.31
	18 to <35 (n=23)	9 (39)	14 (61)				
	35-60 (n=42)	22 (52)	20 (48)				
**Annual average income (CN ¥; US $)**					1.917 (1)		.17
	<120,000 (<16,804; n=31)	12 (39)	19 (61)				
	≥120,000 (≥16,804; n=34)	19 (56)	15 (44)				
**Region**					1.956 (2)		.38
	Eastern China (n=31)	17 (55)	14 (45)				
	Central China (n=14)	7 (50)	7 (50)				
	Western China (n=20)	7 (35)	13 (65)				
**Education**					4.231 (2)		.12
	Master’s and doctoral degrees (n=20)	13 (65)	7 (35)				
	Bachelor’s degree (n=29)	13 (45)	16 (55)				
	Below bachelor’s degree (n=16)	5 (31)	11 (69)				

### Participants’ Experiences of Web-Based Consultations

In total, 31 (48%) out of 65 participants had experience consulting with physicians over the web. During the COVID-19 pandemic, web-based consultations allowed people to avoid going out and minimized the risk of infection. Although web-based consultations were not always feasible with regard to curing diseases, the participants used them as a prediagnosis tool, which helped them make appropriate decisions regarding what to do next about their potential condition. Participant 1 shared his web-based consultation experience with us:

My wife was suffering from gallstones. We paid for an appointment with a famous physician to receive advice on the need for surgery. After uploading the results of an exam and consulting with the physician through the internet, we accepted his suggestion and she underwent an operation.

### Factors Explaining the Use of Web-Based Consultations With Physicians

#### Necessity Analysis of Individual Conditions

The first step of QCA is to examine whether a single condition (including its noncollection) is necessary for a complete merger. When the consistency level is greater than 0.8, the condition is considered sufficient for the use of web-based consultations with physicians. When the consistency level is greater than 0.9, the condition is regarded as necessary for the use [17,20,21].

Table 3 shows the test result of the necessary conditions for the use of web-based consultations with physician using the *fsQCA 3.0* package. The consistency of “complementary role” and “perceived convenience” exceeded 0.9. Thus, the complementary role of web-based consultations and its perceived convenience are necessary conditions for the use (consistency of 0.968 and 0.935, respectively), followed by user’s confidence (consistency of 0.806), which is a sufficient condition for the use.

**Table 3:** Necessity test of the factors associated with use of web-based consultations with physicians.

Condition variable	Use of web-based consultations		~^a^Use of web-based consultations	
Age	0.484	0.517	0.412	0.483
~Age	0.516	0.444	0.588	0.556
Education	0.516	0.464	0.544	0.536
~Education	0.484	0.492	0.456	0.508
Income	0.355	0.611	0.206	0.389
~Income	0.645	0.426	0.794	0.574
Perceived convenience	*0.935* ^b^	0.547	0.706	0.453
~Perceived convenience	0.065	0.167	0.294	0.833
Complementary role	*0.968*	0.698	0.382	0.302
~Complementary role	0.032	0.045	0.618	0.955
User’s confidence	*0.806*	0.758	0.235	0.242
~User’s confidence	0.194	0.188	0.765	0.813

^a^“~” means that a factor does not appear or is “not.”

^b^Italics denote that the consistency exceeded 0.8.

#### Adequacy Analysis of Conditional Configuration

In operating the truth table, the configuration analysis was applied to reveal the sufficiency analysis of the use caused by different configurations composed of multiple conditions. We set the consistency threshold to 0.8 and the case frequency threshold to 1 and calculated the complex solution, parsimonious solution, and intermediate solution.

As indicated in Table 4, there are 4 paths to promote the “use of web-based consultation with physicians.” Among the 4 combined paths, the unique coverage of S2 and S4 was 0.177 and 0.274, respectively. The unique coverage of S1 and S3 was 0.032. In total, these 4 paths showed strong explanatory power due to the good consistency (0.953) and the relative high coverage (0.661).

**Table 4:** Configuration analysis of the factors associated with the use of web-based consultations with physicians (solution consistency=0.953; solution coverage=0.661).

Factor	Configuration				
	Enabling-oriented model			Need-oriented model	
	S1	S2	S3		S4
Age	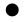 ^a^	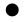	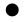		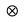 ^b^
Education	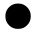 ^c^	—^d^	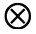 ^e^		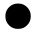
Income	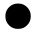	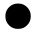	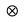		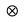
Perceived convenience	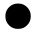	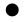	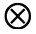		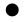
Complementary role	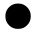	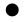	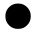		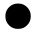
User’s confidence	—	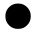	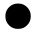		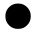
Consistency	1.000	1.000	1.000		0.895
Raw coverage	0.145	0.290	0.032		0.274
Unique coverage	0.032	0.177	0.032		0.274

^a^General conditions.

^b^General conditions do not appear.

^c^Core condition.

^d^Corresponding conditions with path do not matter.

^e^Core conditions do not appear.

Based on the Andersen Behavioral Model [11], we merged the 4 paths into 2 to build a more explanatory model. The first interpretation model is an enabling-oriented model (M1), which includes paths S1 and S2. The basic expression is M1 = age × income × perceived convenience × complementary role (× education + × user’s confidence). The basic combination is the enabling dimension including income and perceived convenience. That is, when web-based consultation brings perceived convenience, the relative high-income group will opt for it.

The participants regarded time saving and avoiding infection during the COVID-19 pandemic as the main conveniences brought by web-based consultations, whereas they regarded complex conduct procedures and late responses as inconveniences.

Participant 2 used web-based consultations because of its time saving characteristic. He said the following:

I have always made appointments with physicians through Haodaifu [an internet health care platform]. I am satisfied with their services because this website informs me about an upcoming appointment beforehand. Meanwhile, the physicians come [for the consultation] on time. The waiting time is not much.

Participant 3 used web-based consultations to avoid COVID-19 infection. She said the following:

I get nervous when my little kid feels any discomfort. On the one hand, I am afraid to go to the hospital because of the risk of infection owing to the COVID-19 pandemic. On the other hand, I also get worried about the adverse consequences of delaying [the child’s treatment]. As a result, I usually opt for a web-based consultation immediately, and use it to determine the necessity of an in-person visit.

Participant 4 complained about the complex procedures that lack instructions. He said the following:

The registration process is complex. A lot of personal information must be entered before beginning the web-based consultation. Due to a lack of clear instructions, it is difficult to figure out how to begin the service. I attempted to register the system, but it was unable to use the service.

The second interpretation model is a need-oriented model (M2), which includes paths S3 and S4. The basic expression is M2 = complementary role × user’s confidence × ~income (× age × ~education × ~perceived convenience + × ~age × education × perceived convenience). The basic combination is the need dimension including complementary role and user’s confidence. That is, regardless of age and education, when web-based consultations are needed, the relative non–high-income group does not care whether it is inconvenient and will opt for it.

In terms of minor problems or primary suggestions, web-based consultations were regarded as complementary to conventional consultations. Participants 3 and 5 said the following, respectively:

I also get worried about the adverse consequences of delaying [the child’s treatment]. As a result, I usually opt for a web-based consultation immediately, and use it to determine the necessity of an in-person visit.

Some specialties, such as dentistry and ophthalmology, require careful examination through the use of instruments before making the diagnosis. Regarding urgent cases, it would still be better for patients to visit the hospital.

Compared with in-person consultations, web-based communications are less smooth because physicians are unable to observe the patient’s body language and emotions. Some participants mentioned that web-based consultation services cannot perform laboratory tests and physical exams when it comes to diagnosis. Moreover, the costs of web-based consultations are not yet covered by the social health care insurance system. This means that patients will have to bear the cost of internet health care services. Due these reasons, some participants did not regard it as a substitute for in-person consultations. Regarding this, Participants 6 and 7 stated the following, respectively:

If it is not face-to-face consultation, I am afraid I could not describe [the symptom] clearly and the doctors would misunderstand me.

For senior physicians of P Hospital, the cost of a web-based consultation is three times that of a conventional consultation. Meanwhile, the expenditure on web-based consultations cannot be reimbursed by social healthcare insurance.

Some participants do not have confidence in web-based consultation services owing to privacy, safety, and qualification concerns, as well as problems surrounding web-based diagnosis. Below is an interview excerpt of Participant 8, who has experience in using text web-based consultations but not video consultations:

Although I never used video consultations, I am afraid that the system records the whole process automatically. I am worried that the video will be misused without my permission. Meanwhile, it is difficult to confirm the qualification of the doctors providing the service. After visiting the professor in C Hospital for a lung infection, I uploaded the results of a chest CT for further suggestions. I doubted the suggestion made by the professor’s students primarily. Given the busy schedule of the professor, his students had made the initial suggestions, which were later checked by the professor himself. So, I only trust the platforms run by public hospitals.

#### Robustness Test

To test the robustness, we increased the consistency level from 0.8 to 0.85 and we also decreased it to 0.72. The result showed that the configuration paths after the adjustment were consistent with those before the adjustment, and the coverage and consistency did not change substantially. Therefore, the results were robust.

## Discussion

### Principal Findings

We examined the current status of the use of web-based consultations with physicians and the factors associated with the service among young and middle-aged Chinese individuals. About half (31/65, 48%) of 18- to 60-year-old residents have experienced web-based consultations. Among the factors associated with the use of web-based consultation, perceived convenience, complementary role, and user’s confidence were found to be the most essential factors.

### Optimizing Web-Based Consultations

We found that perceived convenience is a necessary condition enabling participants to use web-based consultations. In this study, time saving and avoiding COVID-19 infection, which are conveniences provided by web-based consultations, promote the participants’ use of it. Participants in our study, similar to patients in other countries, strongly wish to spend less time to access the services, both when making appointments and while waiting for the appointment at the location; they prefer web-based access to appointment scheduling, want SMS text messaging services for reminders, and prefer for physicians to be available during evenings and weekends [22]. The web-based consultation system provided patients with time-saving and convenient solutions for their health care needs across all treatment processes. Patients could make appointments according to their own schedule and do not have to spend time traveling to the appointments. These findings concur with the research done in the United States. Almathami et al [23] conducted a survey in Saudi Arabia and found that saving time would increase the motivation toward the use of web-based consultations.

The COVID-19 pandemic positively influenced web-based consultation use. This is in line with findings of past studies. Studies show that internet health care services enable patients to avoid going out, decrease the time spent at hospitals when patients need to visit hospitals, and minimize infection risks [3,22,24]. Thus, it is not surprising that the COVID-19 pandemic catalyzed the development and use of the service. Although the service cannot fully substitute traditional in-person appointments, various patients were willing to use web-based consultations in the post–COVID-19 era in the long term.

In our research, participants remarked about the unclear instructions hampering the use of the service. Prior studies also find that patients who lack basic internet-related knowledge are excluded from internet health care services [25,26]. The web-based consultation environment requires patients to be well versed in using web-based platforms and electronic gadgets, and the skill levels regarding this vary by patient. Those with low literacy or limited internet-related knowledge are reluctant to use the service. This potential situation was highlighted in prior studies [27,28]. In the future, web-based consultation providers could attempt to assist persons with less accessibility to the platforms by creating intuitive instructions or even providing staff to support these people and explain how they can navigate the service step-by-step. They can also train patients on the use of available technologies prior to them making an appointment. For example, a video on how to book a web-based consultation could be provided on the front page to guide patients.

### Focusing on the Needs of Residents

We found that once the participants’ needs were met, they opted to use web-based consultations with physicians.

In the study, although web-based consultations do not have provisions for laboratory tests and physical exams, they serve as a supplement for minor issues and primary suggestions.

Meanwhile, some studies reported that web-based consultations improved outcomes in chronic disease management such as diabetes and hyperactivity disorder [29,30]. Considering patients’ preference and need, applying the service in chronic diseases management and subsequent visits may expand its complementary role and benefit patients to a greater extent.

Our results found that some participants did not regard web-based consultations as complementary due to its cost. This is in line with previous studies that found that the cost is a barrier influencing the use of the service, even in high-income countries [26]. Moreover, similar to Germany and the United States, clear regulations about web-based consultations are lacking in China; accordingly, not only do the costs of web-based consultations vary widely, but expenditures on the service are not yet covered by the social health insurance systems [5,31]. Thus, the economic burden on patients may impede their use of web-based consultations.

Users’ confidence is a sufficient factor influencing the use of web-based consultations in the study. The participants use the service if they feel safe. Several participants expressed concerns about the safety and privacy of web-based platforms, as well as the qualification of physicians. This corroborates the findings of prior studies, wherein participants expressed their concern about such safety and privacy issues and believed that the safety and privacy of users should be guaranteed by clear regulations for such services [32,33]. These regulations should ensure that patient data cannot be misused for purposes other than health care or shared without patients’ informed consent. Best practices and standards should also be created to ensure that providers have the relevant qualifications and service quality to provide web-based consultations.

### Limitations

Because of the COVID-19 pandemic, our semistructured interviews were mostly conducted through phone calls. This hindered our ability to observe the participants’ body language and nonverbal cues. Nonetheless, we contacted the participants to explain the topic and purpose of the interview. We shared the questions with the participants 1-7 days in advance, which the participants deemed as adequate and reasonable, enabling them to provide more comprehensive information.

### Conclusions

In conclusion, the Andersen Behavioral Model represents a profound reflection and exploration of the factors associated with web-based consultation use from the user’s perspective. Additionally, the csQCA offers guidance for optimizing the benefits of the service. Perceived convenience, complementary role, and user’s confidence are the essential influencers associated with the use of the service. Clear instructions, comprehensive regulations, and appropriate application can promote the positive development of web-based consultations.

## Abbreviations

csQCA: crisp-set qualitative comparative analysis
M1: enabling-oriented model
M2: need-oriented model
QCA: qualitative comparative analysis

## Data Availability

The data that support the findings presented in this study are available from the corresponding author on reasonable request.

## References

[1] Zhang X, Qing Q, P DZ, T LK (2017). Understanding and analysis of "Internet+Medicine" [Article in Chinese]. China Health Industry.

[2] Del Prete E, Francesconi A, Palermo G, Mazzucchi S, Frosini D, Morganti R, Coleschi P, Raglione LM, Vanni P, Ramat S, Novelli A, Napolitano A, Battisti C, Giuntini M, Rossi C, Menichetti C, Ulivelli M, de Franco V, Rossi S, Bonuccelli U, Ceravolo R (2021). Prevalence and impact of COVID-19 in Parkinson's disease: evidence from a multi-center survey in Tuscany region. J Neurol.

[3] Hassan A, Mari Z, Gatto EM, Cardozo A, Youn J, Okubadejo N, Bajwa JA, Shalash A, Fujioka S, Aldaajani Z, Cubo E (2020). Global survey on telemedicine utilization for movement disorders during the COVID-19 pandemic. Mov Disord.

[4] Seiler N, Chaudhry HJ, Lovitch K, Heyison C, Karacuschansky A, Organick-Lee P, Osei A, Stoll H, Dwyer G, Horton K (2022). Telehealth services and the law: the rapidly evolving regulatory landscape and considerations for sexually transmitted infection and HIV services. Sex Transm Dis.

[5] Byambasuren O, Greenwood H, Bakhit M, Atkins T, Clark J, Scott AM, Glasziou P (2023). Comparison of telephone and video telehealth consultations: systematic review. J Med Internet Res.

[6] Wang W, Sun L, Liu T, Lai T (2022). The use of e-health during the COVID-19 pandemic: a case study in China's Hubei province. Health Sociol Rev.

[7] Zhou C, Hao Y, Lan Y, Li W (2023). To introduce or not? strategic analysis of hospital operations with telemedicine. Eur J Oper Res.

[8] Zhang J (2022). China's internet medical industry summary 2022. Analysys.

[9] Almathami HKY, Win KT, Vlahu-Gjorgievska E (2020). Barriers and facilitators that influence telemedicine-based, real-time, online consultation at patients' homes: systematic literature review. J Med Internet Res.

[10] Li D, Hu Y, Pfaff H, Wang L, Deng L, Lu C, Xia S, Cheng S, Zhu X, Wu X (2020). Determinants of patients' intention to use the online inquiry services provided by internet hospitals: empirical evidence from China. J Med Internet Res.

[11] Andersen R (1968). A Behavioral Model of Families' Use of Health Services.

[12] Lemming MR, Calsyn RJ (2004). Utility of the behavioral model in predicting service utilization by individuals suffering from severe mental illness and homelessness. Community Ment Health J.

[13] Andersen RM (2008). National health surveys and the behavioral model of health services use. Med Care.

[14] Teng L, Li Y (2022). Analysis on the willingness and influencing factors of choosing primary healthcare institutions among patients with chronic conditions in China: a cross-sectional study. BMJ Open.

[15] Zahroh RI, Sutcliffe K, Kneale D, Vazquez Corona M, Betrán AP, Opiyo N, Homer CSE, Bohren MA (2023). Educational interventions targeting pregnant women to optimise the use of caesarean section: what are the essential elements? a qualitative comparative analysis. BMC Public Health.

[16] Ragin CC, Rihous B, Ragin CC (2009). Qualitative comparative analysis using fuzzy sets (fsQCA). Configurational Comparative Methods: Qualitative Comparative Analysis (QCA) and Related Techniques.

[17] Ragin CC (2008). Redesigning Social Inquiry: Fuzzy Sets and Beyond.

[18] Harris K, Kneale D, Lasserson TJ, McDonald VM, Grigg J, Thomas J (2019). School-based self-management interventions for asthma in children and adolescents: a mixed methods systematic review. Cochrane Database Syst Rev.

[19] Farrugia B (2019). WASP (write a scientific paper): an introduction to set-theoretic methods and qualitative comparative analysis. Early Hum Dev.

[20] Thiem A (2017). Standards of good practice and the methodology of necessary conditions in qualitative comparative analysis. Polit Anal.

[21] Lu L, Shi S, Liu B, Liu C (2023). Analysis of factors influencing the organizational capacity of institutional review boards in China: a crisp-set qualitative comparative analysis based on 107 cases. BMC Med Ethics.

[22] Zhang C, Zhu K, Lin Z, Huang P, Pan Y, Sun B, Li D (2021). Utility of deep brain stimulation telemedicine for patients with movement disorders during the COVID outbreak in China. Neuromodulation.

[23] Almathami HKY, Win KT, Vlahu-Gjorgievska E (2022). An empirical study on factors influencing consumers' motivation towards teleconsultation system use. a preliminary report about the Sehha application from Saudi Arabia. Int J Med Inform.

[24] Wosik J, Fudim M, Cameron B, Gellad ZF, Cho A, Phinney D, Curtis S, Roman M, Poon EG, Ferranti J, Katz JN, Tcheng J (2020). Telehealth transformation: COVID-19 and the rise of virtual care. J Am Med Inform Assoc.

[25] Mascaro JS, Catic A, Srivastava M, Diller M, Rana S, Escoffery C, Master V (2022). Examination of provider and patient knowledge, beliefs, and preferences in integrative oncology at a National Cancer Institute-designated comprehensive cancer center. Integr Med Rep.

[26] Neves AL, Burgers J (2022). Digital technologies in primary care: implications for patient care and future research. Eur J Gen Pract.

[27] Neavel C, Watkins SC, Chavez M (2022). Youth, social media, and telehealth: how COVID-19 changed our interactions. Pediatr Ann.

[28] Chen K, Zhang C, Gurley A, Akkem S, Jackson H (2023). Appointment non-attendance for telehealth versus in-person primary care visits at a large public healthcare system. J Gen Intern Med.

[29] Kubes JN, Jones L, Hassan S, Franks N, Wiley Z, Kulshreshtha A (2022). Differences in diabetes control in telemedicine vs. in-person only visits in ambulatory care setting. Prev Med Rep.

[30] Pritchard AE, Northrup RA, Peterson R, Lieb R, Wexler D, Ng R, Kalb L, Ludwig N, Jacobson LA (2023). Can we expand the pool of youth who receive telehealth assessments for ADHD? covariates of service utilization. J Atten Disord.

[31] Dorsey ER, Okun MS, Bloem BR (2020). Care, convenience, comfort, confidentiality, and contagion: the 5 C's that will shape the future of telemedicine. J Parkinsons Dis.

[32] Oelmeier K, Schmitz R, Möllers M, Braun J, Deharde D, Sourouni M, Köster HA, Apsite G, Eveslage M, Fischhuber K, Storck M, Emming F, Wohlmann J, Juhra C (2022). Satisfaction with and feasibility of prenatal counseling via telemedicine: a prospective cohort study. Telemed J E Health.

[33] Chen K, Davoodi NM, Strauss DH, Li M, Jiménez Frances N, Guthrie KM, Goldberg EM (2022). Strategies to ensure continuity of care using telemedicine with older adults during COVID-19: a qualitative study of physicians in primary care and geriatrics. J Appl Gerontol.

